# Supplementary figures and images for: Modeling hormonal control of cambium proliferation
Source: PLoS One. 2017 Feb 10;12(2):e0171927. doi: 10.1371/journal.pone.0171927 (PMC5302410; doi:10.1371/journal.pone.0171927)

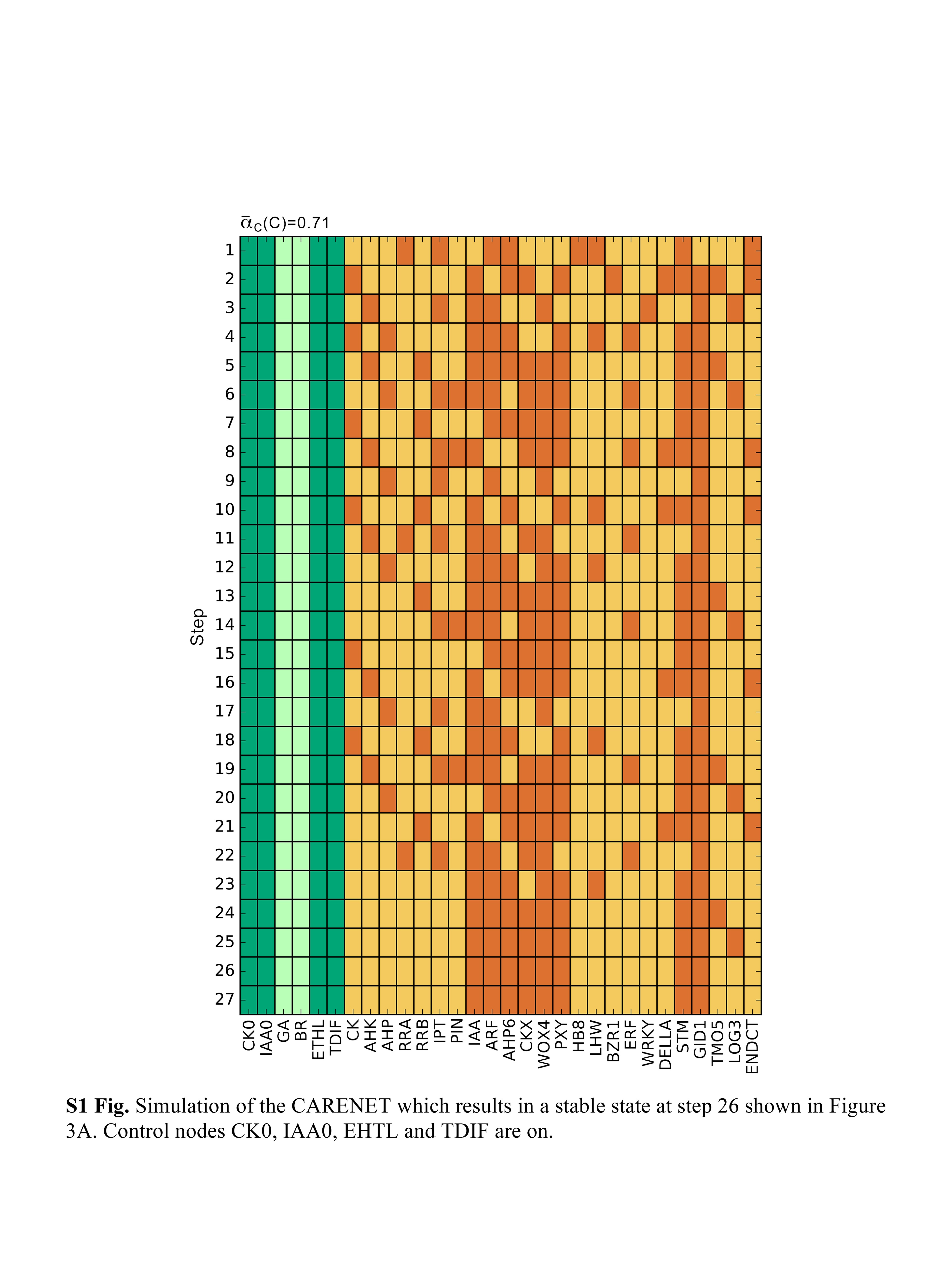

Supplement: S1 Fig — Control nodes CK0, IAA0, EHTL and TDIF are on. (TIF) [file pone.0171927.s001.tif]

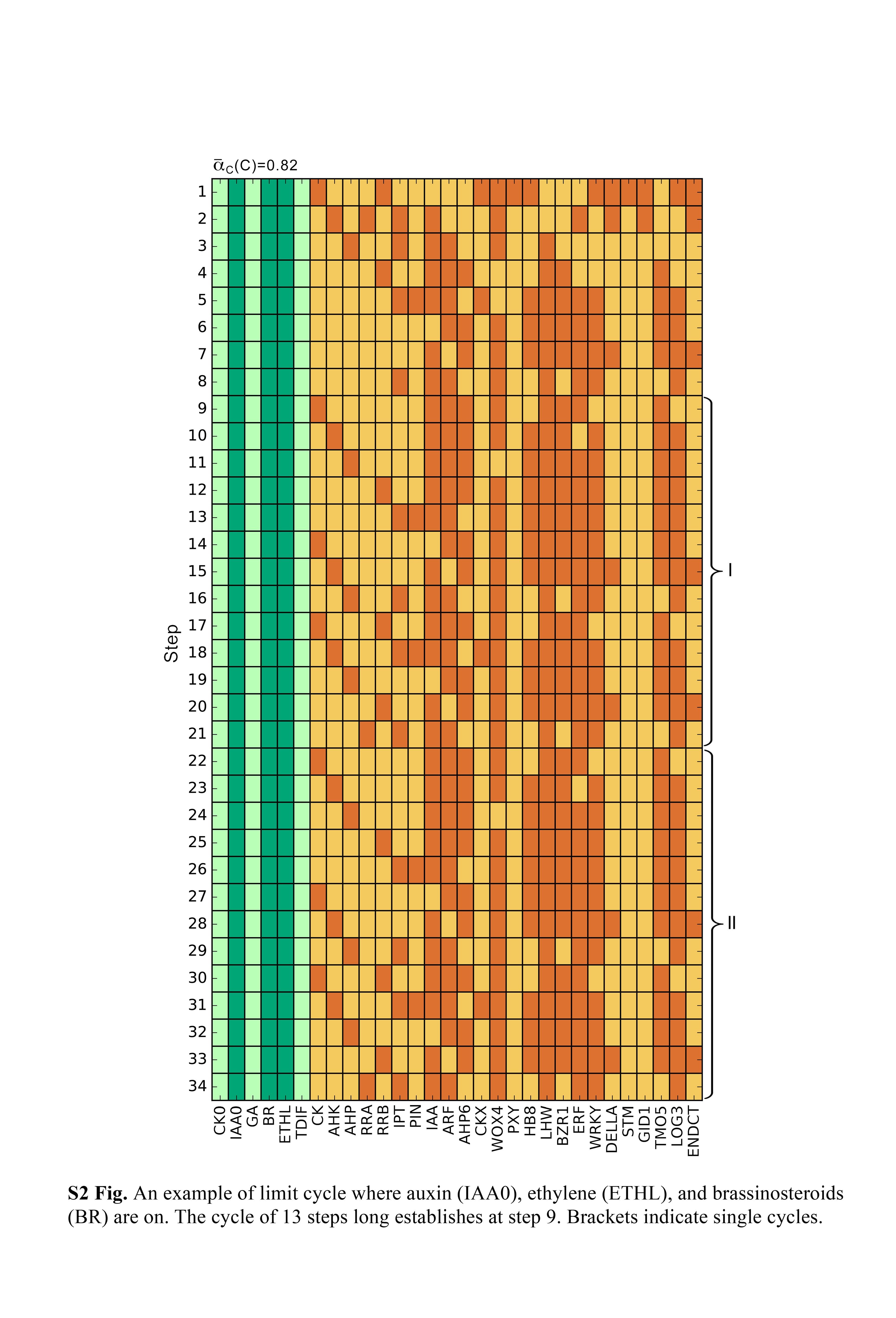

Supplement: S2 Fig — The cycle of 6 steps long establishes at step 9. Brackets indicate single cycles. (TIF) [file pone.0171927.s002.tif]

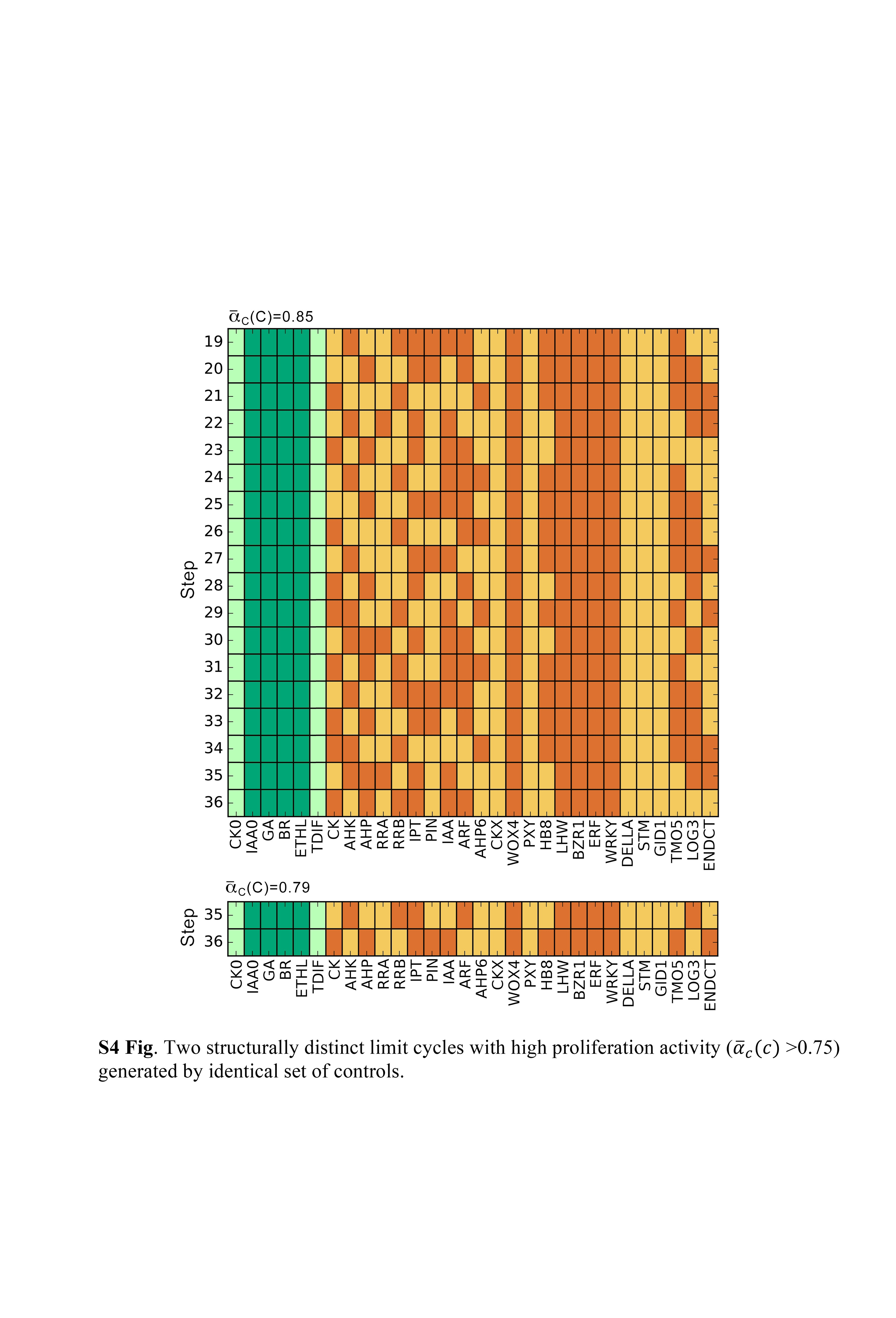

Supplement: S4 Fig — (TIF) [file pone.0171927.s004.tif]
