# Supplementary material for: Modeling hormonal control of cambium proliferation
Source: PLoS One. 2017 Feb 10;12(2):e0171927. doi: 10.1371/journal.pone.0171927 (PMC5302410; doi:10.1371/journal.pone.0171927)
Supplement: S1 Table — The rules are inferred form the experimental evidence shown in S2 Table. (DOCX) [file pone.0171927.s005.docx]

**S1 Table. Rules for calculating the status of each node in the CARENET.** The rules are inferred form the experimental evidence shown in S2 Table.

| **Network node** | **Update rule** |
| --- | --- |
| BR | remains constant through the run |
| CK0 | remains constant through the run |
| ETHL | remains constant through the run |
| GA | remains constant through the run |
| IAA0 | remains constant through the run |
| TDIF | remains constant through the run |
| A-Type ARRs (RRA) | 1 if RRA - ARF > 0  0 else |
| AHK | 1 if CK = 1  0 else |
| AHP6 | 1 if ARF – RRB > 0  0 else |
| AHP | 1 if AHK = 1 and RRA + AHP6 < 2  0 else |
| ARF | 1 if IAA = 1  0 else |
| BIN2 | 1 if BR – PXY < 1  0 else |
| B-Type ARRs (RRB) | 1 if AHP = 1  0 else |
| BZR1 | 1 if BIN2 + DELLA < 1  0 else |
| CK | 1 if CK0 + LOG3 + IPT – CKX > 1  0 else |
| CKX | 1 if ARF + RRB + STM – WRKY > 1  0 else |
| DELLA | 1 if IAA + GA < 1  0 else |
| ENDO | 1 if IAA = 0  0 else |
| ERF | 1 if ETHL = 1 and RRB + WRKY > 0  0 else |
| HB8 (ATHB8) | 1 if ARF + BZR1 > 1  0 else |
| IAA | 1 if PIN = 0 and IAA0 + GA > 0  0 else |
| IPT | 1 if ARF – RRB < 1  0 else |
| LHW | 1 if STM = 0  0 else |
| LOG3 | 1 if TMO5 = 1  0 else |
| PIN polar localization | 1 if RRB – ENDO > 0  0 else |
| PXY | 1 if TDIF + ARF > 1  0 else |
| STM | 1 if ARF – BR > 0  0 else |
| TMO5 | 1 if LHW + ARF > 1  0 else |
| WOX4 | 1 if PXY + ERF > 0  0 else |
| WRKY | 1 if BZR1 = 1  0 else |
