# Supplementary material for: Modeling hormonal control of cambium proliferation
Source: PLoS One. 2017 Feb 10;12(2):e0171927. doi: 10.1371/journal.pone.0171927 (PMC5302410; doi:10.1371/journal.pone.0171927)
Supplement: S2 Table — (DOC) [file pone.0171927.s006.doc]

**S1 Table. Experimental evidence supporting interactions between nodes of CARENET.**

| **Interaction** | **Evidence** | **References** |
| --- | --- | --- |
| AHK → AHP | AHK transfers phosphate residues to AHP as part of multistep phosphorelay activated by cytokinin | (Hwang and Sheen, 2001) |
| AHP6 –| AHP | *ahp6* partially recovers the mutant phenotype of the CK receptor mutant *wol*. | (Mahonen et al., 2006) |
| AHPs→RRB | AHP1, AHP2, and AHP3 interact with ARR1 in yeast two-hybrid assays.  AHP2 interacts with ARR2 and ARR10 in yeast two-hybrid assays. | (Hwang and Sheen, 2001)  (Suzuki et al., 1998) |
| ARF –| IPT | Application of auxin inhibits transcription of *IPT2* | (Tanaka et al., 2006) |
| ARF –| RRA | The *ARR7* and *ARR15* transcripts are reduced in inflorescence stems treated with auxin.  Transcription of *ARR7* and *ARR15* levels is elevated in apices of the *arf5/mp* mutant. | (Zhao et al., 2010) |
| ARF → CKXs | Analysis of *Arabidopsis* microarray gene expression data showed that IAA-activates transcription of *AtCKX1* and *AtCKX6* (2.9- and 7.9-fold respectively). | (Werner et al., 2006) |
| ARF → STM | Ectopic expression of STM rescues *monopteros* (*arf5*) phenotype | (Ckurshumova et al., 2014) |
| ARF(MP)→TMP5 | A basic helix–loop–helix transcription factor TMP5 (TARGET of MONOPTEROS 5) is the direct target of Auxin-dependent transcription factor (ARF5) MONOPTEROS. | (Schlereth et al., 2010) |
| ARF → PXY | Treatment with auxin trafficking inhibitor NPA induced transcription of *ARF1, 4, 5, 11/19,* and *PXY*. | (Suer et al., 2011) |
| ARF→AHP6 | Microarray data show that *AHP6* is downregulated in *mp* mutant seedlings.  Incubation with IAA for 2 hrs causes a 10-fold increase in *AHP6* expression in roots. | (Schlereth et al., 2010)  (Bishopp et al., 2011) |
| ARF→ATHB8 | Expression of PIN1:GFP and *DR5rev:*:GFP precede *ATHB8:*:GUS expression in developing leaves.  *AtHB8* transcription is responsive to auxin. Mutation of a functional auxin response sequence in the *ATHB8* promoter abrogates auxin responsiveness. | (Donner et al., 2009) |
| BR –| STM1 | Transcription of *STM* is suppressed in plants exoressing constitutively active BRI1 and activated in *bri1*. | (Sun et al., 2010) |
| BR–|BIN2 | Phosphorylation of BIN2 by BR effector BRI1 inhibits activity of BIN2. | (Clouse, 2002) |
| BIN2–|BZR1 | Phosphorylation of BZR1 by BIN2 triggers BZR1 degradation. | (Clouse, 2002) |
| BZR1→ATHB8 | In *bri1* the level of ATHB-8 transcription is reduced by 50%.  In the cross of *bri1-116* and dominant-positive *bzr1-1D* the ATHB-8 transcription is rescued by 25%. BZR1 is BRI1-dependent transcription factor. | (Sun et al., 2010) |
| BZR1→WRKY | Y831F dominant-positive BRI1 up-regulates *WRKY48*.  *WRKY12* is down-regulated in *bri1.* | (Oh et al., 2011)  (Sun et al., 2010) |
| CK→AHKs  (as part of the MSP) | AHKs contains a receiver domain.  *AHK2* and *AHK3* complement yeast mutants in a cytokinin-dependent manner.  *AHK4* (*wol/cre*) mutant lacks cytokinin responses.  AHK4 binds active CKs in yeast assays.  Plant carrying different combinations of *AHK2, AHK3* and *AHK4* mutant alleles show CK deficiency phenotypes. | (Hwang and Sheen, 2001)  (D'Agostino et al., 2000)  (Sheen, 2002)  (Suzuki et al., 2001)  (Hutchison et al., 2006; Nishimura et al., 2004) |
| CKX –| CK | Ectopic overexpression of CKXs results in decreased both endogenous CK levels and sensitivity to exogenous CKs.  CKXs (1-7) catalyze the irreversible degradation of CKs. | (Pernisova et al., 2009)  (Bartrina et al., 2011; Werner et al., 2003) |
| DELLA –| BZR1 | Association of DELLA with BZR1 inhibits binding of BZR1 to DNA | (Xu et al., 2013) |
| Endocytosis –| PIN | Endocytosis increases PIN internalization leading to reduction of PIN at the plasma membrane | (Paciorek et al., 2005) |
| ERF→WOX4 | Ethylene over-production mutant *eto1* exhibits enhanced radial growth.  Inhibition of ethylene signaling in *erf109 erf018* double mutant results in transcriptional down-regulation of *WOX4.*  Ethylene promotes secondary growth in poplar | (Etchells et al., 2012)  (Love et al., 2009) |
| Ethylene → ERF | Binding if ethylene to the receptors leads to transcriptional up-regulation of *ERFs*. | (Merchante et al., 2013) |
| GA → GID1 | GID1 becomes activated upon GA binding | (Xu et al., 2013) |
| GA → IAA | GA promotes *PIN1* transcription in proximal xylem cells | (Bjorklund et al., 2007) |
| GID1 –| DELLA | GID1 promotes degradation of DELLA | (Xu et al., 2013) |
| IAA –| DELLA | Auxin inhibits accumulation of DELLA | (Xu et al., 2013) |
| IAA –| Endocytosis | IAA and 2,4-D inhibited Brefeldin A-induced internalization of plasmalemma integral or associated proteins PIN1, PIN2, PIN4, H^+^-ATPase, and water channel PIP2. | (Paciorek et al., 2005) |
| IAA → ARF | IAA de-represses a family of transcription factors ARFs through degradation of IAA/AUX proteins. | (Hardtke and Berleth, 1998; Peer, 2013) |
| IPT → CK | Overexpression of AtIPT4 or AtIPT8 confers cytokinin-independent shoot formation on calli, and overexpression of AtIPT1, 3, 4, 5, 7, or 8 causes increased iP-type cytokinin levels *in planta*.  The rate-limiting step of CK biosynthesis is catalyzed by enzymes encoded by the *IPT* gene family. | (Kamada-Nobusada and Sakakibara, 2009; Sakakibara, 2006; Sun et al., 2003; Werner et al., 2006) |
| LHW → TMO5 | TMO5 and LHW work as a dimer to promote transcription of *LOG3/4*. | (De Rybel et al., 2013; Ohashi-Ito et al., 2014) |
| LOG3/4→CK | LONELY GUY3/4 are isoforms of the enzyme responsible for the final biosynthesis step of cytokinin. | (Kuroha et al., 2009) |
| PIN –| IAA | Accumulation of PIN on the plasma membrane increases auxin efflux from the cells | (Paciorek et al., 2005) |
| PXY → WOX4 | *WOX4* transcript is upregulated rapidly after exogenous application or over-epxression of TDIF in a TDR-dependent manner.  This rapid activation of *WOX4* expression is not observed in the *tdr-1* mutant (also known as *pxy-5*). | (Etchells et al., 2013; Hirakawa et al., 2010) |
| PXY → BIN2 | PXY interacts with GSK3s (BIN2) at the plasma membrane and activates BIN2 in a TDIF-dependent manner | (Kondo et al., 2014) |
| RRA –| AHP | Higher order mutants of *RRA* (A-type Arabidopsis Response Regulator) show increased sensitivity to CK.  Spatial patterns of *RRA* transcription and CK response are consistent with partially redundant function of these genes in CK signaling. RRA decreases *RRB6*::LUC. | (Hwang and Sheen, 2001; To et al., 2007) |
| RRB –| AHP6  RRB → IPT | Both links were proposed by Benitez and Hejatko as an essential interaction that maintains identity of xylem, cambium, and phloem. | (Benitez and Hejatko, 2013) |
| RRB → CKX | Ectopic expression of constitutively active ARR2 promotes transcription of cytokinin oxidase *CKX3* gene (AT5G56970) | (Hass et al., 2004) |
| RRB → ERF | ARR2 binds to *ERF1* promoter and ectopic expression of ARR2 causes 20 folds up-regulation of *ERF1* promoter activity. Transcription of *ERF1* is reduced in *arr2*. | (Hass et al., 2004) |
| RRB → RRA | Constitutively active RRB promotes transcription of *RRA.* | (Hass et al., 2004) |
| RRB→PIN | PIN transcription is inhibited in roots treated with cytokinin; the level of PIN in a *rrb* mutant allele upon treatment with cytokinin remains higher than in the wild type plants.  RRB stimulates vacuolar degradation of PIN | ⁠(Dello Ioio et al., 2008)  (Marhavy et al., 2011) |
| STM –| LHW | Over-expression of Poplar homologue of STM (ARBORNOX1 ) inhibits transcription of *LHW* | (Liu et al., 2015) |
| STM → CKX | Over-expression of Poplar homologue of STM (ARBORNOX1) promotes transcription of *Cytokinin Oxidase 5.* | (Liu et al., 2015) |
| TDIF → PXY | Small peptides of the CLE family (CLE41, 44 and CLE42) act as tracheary elements differentiation inhibitors in both *Zinnia* and *Arabidopsis*.  Plants homozygous for *tdr* are insensitive to TDIF.  TDIF binds to the leucine-reach repeats of PXY | (Etchells and Turner, 2010; Hirakawa et al., 2008; Ito et al., 2006; Whitford et al., 2008)  (Zhang et al., 2016) |
| TMO5 → LOG3 | Dimer of TMO5-LHW promote transcription of *LOG3* and *LOG4*. | (De Rybel et al., 2013) |
| WRKY –| CKX | Transcription of *CKX* is up-regulated in two *wrky12* knock-down alleles. | (Wang et al., 2010) |
| WRKY → ERF | ERF4 gene is down-regulated in a *wrky* allele of *Medicago truncatula* *WRKY12* homologue. | (Wang et al., 2010) |

**References**

Bartrina, I., E. Otto, M. Strnad, T. Werner, and T. Schmulling. 2011. Cytokinin Regulates the Activity of Reproductive Meristems, Flower Organ Size, Ovule Formation, and Thus Seed Yield in Arabidopsis thaliana. *The Plant cell*. 23:69-80.

Benitez, M., and J. Hejatko. 2013. Dynamics of cell-fate determination and patterning in the vascular bundles of Arabidopsis thaliana. *PloS one*. 8:e63108.

Bishopp, A., H. Help, S. El-Showk, D. Weijers, B. Scheres, J. Friml, E. Benkova, A.P. Mahonen, and Y. Helariutta. 2011. A mutually inhibitory interaction between auxin and cytokinin specifies vascular pattern in roots. *Current biology : CB*. 21:917-926.

Bjorklund, S., H. Antti, I. Uddestrand, T. Moritz, and B. Sundberg. 2007. Cross-talk between gibberellin and auxin in development of Populus wood: gibberellin stimulates polar auxin transport and has a common transcriptome with auxin. *The Plant journal : for cell and molecular biology*. 52:499-511.

Ckurshumova, W., T. Smirnova, D. Marcos, Y. Zayed, and T. Berleth. 2014. Irrepressible MONOPTEROS/ARF5 promotes de novo shoot formation. *The New phytologist*. 204:556-566.

Clouse, S.D. 2002. Brassinosteroid signal transduction: Clarifying the pathway from ligand perception to gene expression. *Molecular cell*. 10:973-982.

D'Agostino, I.B., J. Deruere, and J.J. Kieber. 2000. Characterization of the response of the arabidopsis response regulator gene family to cytokinin. *Plant physiology*. 124:1706-1717.

De Rybel, B., B. Moller, S. Yoshida, I. Grabowicz, P. Barbier de Reuille, S. Boeren, R.S. Smith, J.W. Borst, and D. Weijers. 2013. A bHLH complex controls embryonic vascular tissue establishment and indeterminate growth in Arabidopsis. *Developmental cell*. 24:426-437.

Dello Ioio, R., K. Nakamura, L. Moubayidin, S. Perilli, M. Taniguchi, M.T. Morita, T. Aoyama, P. Costantino, and S. Sabatini. 2008. A Genetic Framework for the Control of Cell Division and Differentiation in the Root Meristem. *Science*. 322:1380-1384.

Donner, T.J., I. Sherr, and E. Scarpella. 2009. Regulation of preprocambial cell state acquisition by auxin signaling in Arabidopsis leaves. *Development*. 136:3235-3246.

Etchells, J.P., C.M. Provost, L. Mishra, and S.R. Turner. 2013. WOX4 and WOX14 act downstream of the PXY receptor kinase to regulate plant vascular proliferation independently of any role in vascular organisation. *Development*. 140:2224-2234.

Etchells, J.P., C.M. Provost, and S.R. Turner. 2012. Plant vascular cell division is maintained by an interaction between PXY and ethylene signalling. *PLoS genetics*. 8:e1002997.

Etchells, J.P., and S.R. Turner. 2010. The PXY-CLE41 receptor ligand pair defines a multifunctional pathway that controls the rate and orientation of vascular cell division. *Development*. 137:767-774.

Hardtke, C.S., and T. Berleth. 1998. The Arabidopsis gene MONOPTEROS encodes a transcription factor mediating embryo axis formation and vascular development. 1405-1411 pp.

Hass, C., J. Lohrmann, V. Albrecht, U. Sweere, F. Hummel, S.D. Yoo, I. Hwang, T. Zhu, E. Schafer, J. Kudla, and K. Harter. 2004. The response regulator 2 mediates ethylene signalling and hormone signal integration in Arabidopsis. *Embo Journal*. 23:3290-3302.

Hirakawa, Y., Y. Kondo, and H. Fukuda. 2010. TDIF Peptide Signaling Regulates Vascular Stem Cell Proliferation via the WOX4 Homeobox Gene in Arabidopsis. *The Plant cell*. 22:2618-2629.

Hirakawa, Y., H. Shinohara, Y. Kondo, A. Inoue, I. Nakanomyo, M. Ogawa, S. Sawa, K. Ohashi-Ito, Y. Matsubayashi, and H. Fukuda. 2008. Non-cell-autonomous control of vascular stem cell fate by a CLE peptide/receptor system. *Proceedings of the National Academy of Sciences of the United States of America*. 105:15208-15213.

Hutchison, C.E., J. Li, C. Argueso, M. Gonzalez, E. Lee, M.W. Lewis, B.B. Maxwell, T.D. Perdue, G.E. Schaller, J.M. Alonso, J.R. Ecker, and J.J. Kieber. 2006. The Arabidopsis histidine phosphotransfer proteins are redundant positive regulators of cytokinin signaling. *The Plant cell*. 18:3073-3087.

Hwang, I., and J. Sheen. 2001. Two-component circuitry in Arabidopsis cytokinin signal transduction. *Nature*. 413:383-389.

Ito, Y., I. Nakanomyo, H. Motose, K. Iwamoto, S. Sawa, N. Dohmae, and H. Fukuda. 2006. Dodeca-CLE peptides as suppressors of plant stem cell differentiation. *Science*. 313:842-845.

Kamada-Nobusada, T., and H. Sakakibara. 2009. Molecular basis for cytokinin biosynthesis. *Phytochemistry*. 70:444-449.

Kondo, Y., T. Ito, H. Nakagami, Y. Hirakawa, M. Saito, T. Tamaki, K. Shirasu, and H. Fukuda. 2014. Plant GSK3 proteins regulate xylem cell differentiation downstream of TDIF-TDR signalling. *Nature communications*. 5:3504.

Kuroha, T., H. Tokunaga, M. Kojima, N. Ueda, T. Ishida, S. Nagawa, H. Fukuda, K. Sugimoto, and H. Sakakibara. 2009. Functional Analyses of LONELY GUY Cytokinin-Activating Enzymes Reveal the Importance of the Direct Activation Pathway in Arabidopsis. *The Plant cell*. 21:3152-3169.

Liu, L., M. Zinkgraf, H.E. Petzold, E.P. Beers, V. Filkov, and A. Groover. 2015. The Populus ARBORKNOX1 homeodomain transcription factor regulates woody growth through binding to evolutionarily conserved target genes of diverse function. *The New phytologist*. 205:682-694.

Love, J., S. Bjorklund, J. Vahala, M. Hertzberg, J. Kangasjarvi, and B. Sundberg. 2009. Ethylene is an endogenous stimulator of cell division in the cambial meristem of Populus. *Proceedings of the National Academy of Sciences of the United States of America*. 106:5984-5989.

Mahonen, A.P., A. Bishopp, M. Higuchi, K.M. Nieminen, K. Kinoshita, K. Tormakangas, Y. Ikeda, A. Oka, T. Kakimoto, and Y. Helariutta. 2006. Cytokinin signaling and its inhibitor AHP6 regulate cell fate during vascular development. *Science*. 311:94-98.

Marhavy, P., A. Bielach, L. Abas, A. Abuzeineh, J. Duclercq, H. Tanaka, M. Parezova, J. Petrasek, J. Friml, J. Kleine-Vehn, and E. Benkova. 2011. Cytokinin modulates endocytic trafficking of PIN1 auxin efflux carrier to control plant organogenesis. *Developmental cell*. 21:796-804.

Merchante, C., J.M. Alonso, and A.N. Stepanova. 2013. Ethylene signaling: simple ligand, complex regulation. *Current opinion in plant biology*. 16:554-560.

Nishimura, C., Y. Ohashi, S. Sato, T. Kato, S. Tabata, and C. Ueguchi. 2004. Histidine kinase homologs that act as cytokinin receptors possess overlapping functions in the regulation of shoot and root growth in Arabidopsis. *The Plant cell*. 16:1365-1377.

Oh, M.H., J. Sun, D.H. Oh, R.E. Zielinski, S.D. Clouse, and S.C. Huber. 2011. Enhancing Arabidopsis leaf growth by engineering the BRASSINOSTEROID INSENSITIVE1 receptor kinase. *Plant physiology*. 157:120-131.

Ohashi-Ito, K., M. Saegusa, K. Iwamoto, Y. Oda, H. Katayama, M. Kojima, H. Sakakibara, and H. Fukuda. 2014. A bHLH complex activates vascular cell division via cytokinin action in root apical meristem. *Current biology : CB*. 24:2053-2058.

Paciorek, T., E. Zazimalova, N. Ruthardt, J. Petrasek, Y.D. Stierhof, J. Kleine-Vehn, D.A. Morris, N. Emans, G. Jurgens, N. Geldner, and J. Friml. 2005. Auxin inhibits endocytosis and promotes its own efflux from cells. *Nature*. 435:1251-1256.

Peer, W.A. 2013. From perception to attenuation: auxin signalling and responses. *Current opinion in plant biology*. 16:561-568.

Pernisova, M., P. Klima, J. Horak, M. Valkova, J. Malbeck, P. Soucek, P. Reichman, K. Hoyerova, J. Dubova, J. Friml, E. Zazimalova, and J. Hejatko. 2009. Cytokinins modulate auxin-induced organogenesis in plants via regulation of the auxin efflux. *Proceedings of the National Academy of Sciences of the United States of America*. 106:3609-3614.

Sakakibara, H. 2006. Cytokinins: Activity, biosynthesis, and translocation. *In* Annual review of plant biology. Vol. 57. 431-449.

Schlereth, A., B. Moller, W. Liu, M. Kientz, J. Flipse, E.H. Rademacher, M. Schmid, G. Jurgens, and D. Weijers. 2010. MONOPTEROS controls embryonic root initiation by regulating a mobile transcription factor. *Nature*. 464:913-916.

Sheen, J. 2002. Phosphorelay and transcription control in cytokinin signal transduction. *Science*. 296:1650-1652.

Suer, S., J. Agusti, P. Sanchez, M. Schwarz, and T. Greb. 2011. WOX4 imparts auxin responsiveness to cambium cells in Arabidopsis. *The Plant cell*. 23:3247-3259.

Sun, J.Q., Q.W. Niu, P. Tarkowski, B.L. Zheng, D. Tarkowska, G. Sandberg, N.H. Chua, and J.R. Zuo. 2003. The Arabidopsis AtIPT8/PGA22 gene encodes an isopentenyl transferase that is involved in de novo cytokinin biosynthesis. *Plant physiology*. 131:167-176.

Sun, Y., X.Y. Fan, D.M. Cao, W. Tang, K. He, J.Y. Zhu, J.X. He, M.Y. Bai, S. Zhu, E. Oh, S. Patil, T.W. Kim, H. Ji, W.H. Wong, S.Y. Rhee, and Z.Y. Wang. 2010. Integration of brassinosteroid signal transduction with the transcription network for plant growth regulation in Arabidopsis. *Developmental cell*. 19:765-777.

Suzuki, T., A. Imamura, C. Ueguchi, and T. Mizuno. 1998. Histidine-containing phosphotransfer (HPt) signal transducers implicated in His-to-Asp phosphorelay in Arabidopsis. *Plant and Cell Physiology*. 39:1258-1268.

Suzuki, T., K. Sakurai, C. Ueguchi, and T. Mizuno. 2001. Two types of putative nuclear factors that physically interact with histidine-containing phosphotransfer (Hpt) domains, signaling mediators in His-to-Asp phosphorelay, in Arabidopsis thaliana. *Plant and Cell Physiology*. 42:37-45.

Tanaka, M., K. Takei, M. Kojima, H. Sakakibara, and H. Mori. 2006. Auxin controls local cytokinin biosynthesis in the nodal stem in apical dominance. *The Plant journal : for cell and molecular biology*. 45:1028-1036.

To, J.P., J. Deruere, B.B. Maxwell, V.F. Morris, C.E. Hutchison, F.J. Ferreira, G.E. Schaller, and J.J. Kieber. 2007. Cytokinin regulates type-A Arabidopsis Response Regulator activity and protein stability via two-component phosphorelay. *The Plant cell*. 19:3901-3914.

Wang, H., U. Avci, J. Nakashima, M.G. Hahn, F. Chen, and R.A. Dixon. 2010. Mutation of WRKY transcription factors initiates pith secondary wall formation and increases stem biomass in dicotyledonous plants. *Proceedings of the National Academy of Sciences of the United States of America*. 107:22338-22343.

Werner, T., I. Kollmer, I. Bartrina, K. Holst, and T. Schmulling. 2006. New insights into the biology of cytokinin degradation. *Plant Biol (Stuttg)*. 8:371-381.

Werner, T., V. Motyka, V. Laucou, R. Smets, H. Van Onckelen, and T. Schmulling. 2003. Cytokinin-deficient transgenic Arabidopsis plants show multiple developmental alterations indicating opposite functions of cytokinins in the regulation of shoot and root meristem activity. *The Plant cell*. 15:2532-2550.

Whitford, R., A. Fernandez, R. De Groodt, E. Ortega, and P. Hilson. 2008. Plant CLE peptides from two distinct functional classes synergistically induce division of vascular cells. *Proceedings of the National Academy of Sciences of the United States of America*. 105:18625-18630.

Xu, P., Y. Kong, X. Li, and L. Li. 2013. Identification of molecular processes needed for vascular formation through transcriptome analysis of different vascular systems. *BMC genomics*. 14:217.

Zhang, H., X. Lin, Z. Han, L.J. Qu, and J. Chai. 2016. Crystal structure of PXY-TDIF complex reveals a conserved recognition mechanism among CLE peptide-receptor pairs. *Cell research*. 26:543-555.

Zhao, Z., S.U. Andersen, K. Ljung, K. Dolezal, A. Miotk, S.J. Schultheiss, and J.U. Lohmann. 2010. Hormonal control of the shoot stem-cell niche. *Nature*. 465:1089-1092.
