# Supplementary material for: Modeling hormonal control of cambium proliferation
Source: PLoS One. 2017 Feb 10;12(2):e0171927. doi: 10.1371/journal.pone.0171927 (PMC5302410; doi:10.1371/journal.pone.0171927)
Supplement: S3 Table — (DOCX) [file pone.0171927.s007.docx]

**S3 Table.** Statistical analysis of all stable states generated by the CARENET.

| States of the control nodes | | | | | | Performance | | | Number  of final states | Number of final states with L steps | | | | | | | |
| --- | --- | --- | --- | --- | --- | --- | --- | --- | --- | --- | --- | --- | --- | --- | --- | --- | --- |
| CK0 | IAA0 | GA | BR | ETHL | TDIF | WOX4 | ATHB-8 | $\bar{\alpha}_{c}(c)$ |  | L=1 | L=2 | L=4 | L=5 | L=6 | L=8 | L=13 | L=18 |
| 0 | 0 | 0 | 0 | 0 | 0 | 0 | 0 | 0.000 | 1 | 1 |  |  |  |  |  |  |  |
| 0 | 0 | 0 | 0 | 0 | 1 | 0 | 0 | 0.000 | 1 | 1 |  |  |  |  |  |  |  |
| 0 | 0 | 0 | 0 | 1 | 0 | 0 | 0 | 0.000 | 1 | 1 |  |  |  |  |  |  |  |
| 0 | 0 | 0 | 0 | 1 | 1 | 0 | 0 | 0.000 | 1 | 1 |  |  |  |  |  |  |  |
| 0 | 0 | 0 | 1 | 0 | 0 | 0 | 0 | 0.000 | 1 | 1 |  |  |  |  |  |  |  |
| 0 | 0 | 0 | 1 | 0 | 1 | 0 | 0 | 0.000 | 1 | 1 |  |  |  |  |  |  |  |
| 0 | 0 | 0 | 1 | 1 | 0 | 0 | 0 | 0.000 | 1 | 1 |  |  |  |  |  |  |  |
| 0 | 0 | 0 | 1 | 1 | 1 | 0 | 0 | 0.000 | 1 | 1 |  |  |  |  |  |  |  |
| 0 | 0 | 1 | 0 | 0 | 0 | 0 | 0 | 0.000 | 1 | 1 |  |  |  |  |  |  |  |
| 0 | 0 | 1 | 0 | 0 | 1 | 1 | 0 | 0.707 | 1 | 1 |  |  |  |  |  |  |  |
| 0 | 0 | 1 | 0 | 1 | 0 | 0 | 0 | 0.000 | 1 | 1 |  |  |  |  |  |  |  |
| 0 | 0 | 1 | 0 | 1 | 1 | 1 | 0 | 0.707 | 1 | 1 |  |  |  |  |  |  |  |
| 0 | 0 | 1 | 1 | 0 | 0 | 0 | 0.782 | 0.553 | 7 | 1 | 1 | 1 | 1 | 1 | 1 |  | 1 |
| 0 | 0 | 1 | 1 | 0 | 1 | 0.859 | 0.141 | 0.616 | 7 | 1 | 1 | 1 |  | 1 | 3 |  |  |
| 0 | 0 | 1 | 1 | 1 | 0 | 1 | 0.782 | 0.898 | 7 | 1 | 1 | 1 | 1 | 1 | 1 |  | 1 |
| 0 | 0 | 1 | 1 | 1 | 1 | 0.887 | 0.141 | 0.635 | 7 | 1 | 1 | 1 |  | 1 | 3 |  |  |
| 0 | 1 | 0 | 0 | 0 | 0 | 0 | 0 | 0.000 | 1 | 1 |  |  |  |  |  |  |  |
| 0 | 1 | 0 | 0 | 0 | 1 | 1 | 0 | 0.707 | 1 | 1 |  |  |  |  |  |  |  |
| 0 | 1 | 0 | 0 | 1 | 0 | 0 | 0 | 0.000 | 1 | 1 |  |  |  |  |  |  |  |
| 0 | 1 | 0 | 0 | 1 | 1 | 1 | 0 | 0.707 | 1 | 1 |  |  |  |  |  |  |  |
| 0 | 1 | 0 | 1 | 0 | 0 | 0 | 0.655 | 0.463 | 6 | 1 | 1 | 1 |  |  | 1 | 2 |  |
| 0 | 1 | 0 | 1 | 0 | 1 | 0.862 | 0.109 | 0.614 | 6 | 1 | 1 | 1 |  |  | 3 |  |  |
| 0 | 1 | 0 | 1 | 1 | 0 | 0.899 | 0.655 | 0.787 | 6 | 1 | 1 | 1 |  |  | 1 | 2 |  |
| 0 | 1 | 0 | 1 | 1 | 1 | 0.878 | 0.109 | 0.626 | 6 | 1 | 1 | 1 |  |  | 3 |  |  |
| 0 | 1 | 1 | 0 | 0 | 0 | 0 | 0 | 0.000 | 1 | 1 |  |  |  |  |  |  |  |
| 0 | 1 | 1 | 0 | 0 | 1 | 1 | 0 | 0.707 | 1 | 1 |  |  |  |  |  |  |  |
| 0 | 1 | 1 | 0 | 1 | 0 | 0 | 0 | 0.000 | 1 | 1 |  |  |  |  |  |  |  |
| 0 | 1 | 1 | 0 | 1 | 1 | 1 | 0 | 0.707 | 1 | 1 |  |  |  |  |  |  |  |
| 0 | 1 | 1 | 1 | 0 | 0 | 0 | 0.782 | 0.553 | 7 | 1 | 1 | 1 | 1 | 1 | 1 |  | 1 |
| 0 | 1 | 1 | 1 | 0 | 1 | 0.859 | 0.141 | 0.616 | 7 | 1 | 1 | 1 |  | 1 | 3 |  |  |
| 0 | 1 | 1 | 1 | 1 | 0 | 1 | 0.782 | 0.898 | 7 | 1 | 1 | 1 | 1 | 1 | 1 |  | 1 |
| 0 | 1 | 1 | 1 | 1 | 1 | 0.887 | 0.141 | 0.635 | 7 | 1 | 1 | 1 |  | 1 | 3 |  |  |
| 1 | 0 | 0 | 0 | 0 | 0 | 0 | 0 | 0.000 | 1 | 1 |  |  |  |  |  |  |  |
| 1 | 0 | 0 | 0 | 0 | 1 | 0 | 0 | 0.000 | 1 | 1 |  |  |  |  |  |  |  |
| 1 | 0 | 0 | 0 | 1 | 0 | 1 | 0 | 0.707 | 1 | 1 |  |  |  |  |  |  |  |
| 1 | 0 | 0 | 0 | 1 | 1 | 1 | 0 | 0.707 | 1 | 1 |  |  |  |  |  |  |  |
| 1 | 0 | 0 | 1 | 0 | 0 | 0 | 0 | 0.000 | 1 | 1 |  |  |  |  |  |  |  |
| 1 | 0 | 0 | 1 | 0 | 1 | 0 | 0 | 0.000 | 1 | 1 |  |  |  |  |  |  |  |
| 1 | 0 | 0 | 1 | 1 | 0 | 1 | 0 | 0.707 | 1 | 1 |  |  |  |  |  |  |  |
| 1 | 0 | 0 | 1 | 1 | 1 | 1 | 0 | 0.707 | 1 | 1 |  |  |  |  |  |  |  |
| 1 | 0 | 1 | 0 | 0 | 0 | 0 | 0 | 0.000 | 6 | 1 | 1 | 1 |  |  | 3 |  |  |
| 1 | 0 | 1 | 0 | 0 | 1 | 0.868 | 0 | 0.614 | 6 | 1 | 1 | 1 |  |  | 3 |  |  |
| 1 | 0 | 1 | 0 | 1 | 0 | 0.142 | 0 | 0.100 | 6 | 1 | 1 | 1 |  |  | 3 |  |  |
| 1 | 0 | 1 | 0 | 1 | 1 | 0.879 | 0 | 0.622 | 6 | 1 | 1 | 1 |  |  | 3 |  |  |
| 1 | 0 | 1 | 1 | 0 | 0 | 0 | 0.5 | 0.354 | 2 |  | 1 |  |  | 1 |  |  |  |
| 1 | 0 | 1 | 1 | 0 | 1 | 0.5 | 0.5 | 0.500 | 2 |  | 1 |  |  | 1 |  |  |  |
| 1 | 0 | 1 | 1 | 1 | 0 | 1 | 0.5 | 0.791 | 2 |  | 1 |  |  | 1 |  |  |  |
| 1 | 0 | 1 | 1 | 1 | 1 | 0.884 | 0.5 | 0.718 | 2 |  | 1 |  |  | 1 |  |  |  |
| 1 | 1 | 0 | 0 | 0 | 0 | 0 | 0 | 0.000 | 6 | 1 | 1 | 1 |  |  | 3 |  |  |
| 1 | 1 | 0 | 0 | 0 | 1 | 0.868 | 0 | 0.614 | 6 | 1 | 1 | 1 |  |  | 3 |  |  |
| 1 | 1 | 0 | 0 | 1 | 0 | 0.142 | 0 | 0.100 | 6 | 1 | 1 | 1 |  |  | 3 |  |  |
| 1 | 1 | 0 | 0 | 1 | 1 | 0.879 | 0 | 0.622 | 6 | 1 | 1 | 1 |  |  | 3 |  |  |
| 1 | 1 | 0 | 1 | 0 | 0 | 0 | 0.236 | 0.167 | 2 |  | 1 |  |  | 1 |  |  |  |
| 1 | 1 | 0 | 1 | 0 | 1 | 0.5 | 0.23 | 0.389 | 2 |  | 1 |  |  | 1 |  |  |  |
| 1 | 1 | 0 | 1 | 1 | 0 | 0.882 | 0.236 | 0.646 | 2 |  | 1 |  |  | 1 |  |  |  |
| 1 | 1 | 0 | 1 | 1 | 1 | 0.73 | 0.23 | 0.541 | 2 |  | 1 |  |  | 1 |  |  |  |
| 1 | 1 | 1 | 0 | 0 | 0 | 0 | 0 | 0.000 | 6 | 1 | 1 | 1 |  |  | 3 |  |  |
| 1 | 1 | 1 | 0 | 0 | 1 | 0.868 | 0 | 0.614 | 6 | 1 | 1 | 1 |  |  | 3 |  |  |
| 1 | 1 | 1 | 0 | 1 | 0 | 0.142 | 0 | 0.100 | 6 | 1 | 1 | 1 |  |  | 3 |  |  |
| 1 | 1 | 1 | 0 | 1 | 1 | 0.879 | 0 | 0.622 | 6 | 1 | 1 | 1 |  |  | 3 |  |  |
| 1 | 1 | 1 | 1 | 0 | 0 | 0 | 0.5 | 0.354 | 2 |  | 1 |  |  | 1 |  |  |  |
| 1 | 1 | 1 | 1 | 0 | 1 | 0.5 | 0.5 | 0.500 | 2 |  | 1 |  |  | 1 |  |  |  |
| 1 | 1 | 1 | 1 | 1 | 0 | 1 | 0.5 | 0.791 | 2 |  | 1 |  |  | 1 |  |  |  |
| 1 | 1 | 1 | 1 | 1 | 1 | 0.884 | 0.5 | 0.718 | 2 |  | 1 |  |  | 1 |  |  |  |
